# Hiding in Plain Sight: Human Gingival Fibroblasts as an Essential, Yet Overlooked, Tool in Regenerative Medicine

**DOI:** 10.3390/cells12162021

**Published:** 2023-08-08

**Authors:** Asmaa Fadl, Andrew Leask

**Affiliations:** College of Dentistry, University of Saskatchewan, 105 Wiggins Road, Saskatoon, SK S7N 5A2, Canada; asmaa.fadl@usask.ca

**Keywords:** regenerative repair, fibroblast, mesenchymal progenitor cells, gingiva, wound healing, scarless repair, pluripotency, tissue engineering

## Abstract

Adult human gingival fibroblasts (HGFs), the most abundant cells in the oral cavity, are essential for maintaining oral homeostasis. Compared with other tissues, adult oral mucosal wounds heal regeneratively, without scarring. Relative to fibroblasts from other locations, HGFs are relatively refractory to myofibroblast differentiation, immunomodulatory, highly regenerative, readily obtained via minimally invasive procedures, easily and rapidly expanded in vitro, and highly responsive to growth factors and cytokines. Consequently, HGFs might be a superior, yet perhaps underappreciated, source of adult mesenchymal progenitor cells to use in tissue engineering and regeneration applications, including the treatment of fibrotic auto-immune connective tissue diseases such as scleroderma. Herein, we highlight in vitro and translational studies that have investigated the regenerative and differentiation potential of HGFs, with the objective of outlining current limitations and inspiring future research that could facilitate translating the regenerative potential of HGFs into the clinic.

## 1. Introduction

Fibroblasts, a type of mesenchymal cell, are the most common connective tissue cells. Fibroblasts are responsible for producing extracellular matrix (ECM) components, and, accordingly, play a critical role in promoting the structural integrity, mechanical properties, and repair of tissues [1]. Although fibroblasts from different anatomical sites have essentially identical morphology, DNA-microarray studies have revealed that different types of fibroblasts have their own gene-expression profile and distinct behaviors, based on their location within the body and even within the same organ [1,2,3]. Consequently, fibroblasts from different anatomical locations may act differently based on the function demanded of them.

Regenerative medicine is an emerging field that focuses on the development of innovative therapies to repair or replace damaged tissues and organs [4]. Identifying the ideal cell source to use for tissue regeneration is essential. Embryonic stem cells (ESCs) and fetal stem cells (FSCs) have been considered the most powerful stem cells sources for tissue regeneration; ESCs are pluripotent and possess unlimited self-renewal potential [5]. FSCs, similarly, are significantly more multipotent than their adult counterparts [6]. However, the relatively limited accessibility and ethical controversies associated with employing embryonic or fetal tissue has restricted their use in stem cell research and limited their potential use in tissue engineering and regenerative medicine [7]. Thus, finding an alternative source of stem/progenitor cells that share features comparable to those of fetal stem cells is crucial.

One such source of stem cells may be adult gingival tissue. Both adult gingival and fetal tissue heal without scarring and contain highly regenerative cells with multipotent differentiation capacities [8,9]. The most abundant cells in the adult gingiva, gingival fibroblasts (GFs) [10], are potentially highly important for regenerative medicine. First, GFs have stem/progenitor cell-like multipotent differentiation potential, which has been linked to their embryonic neural crest origin [8]. For example, GFs can differentiate into multiple cell lineages, such as osteoblasts, chondrocytes, and adipocytes, indicating their potential for tissue engineering applications [10]. Importantly, as gingivae heal regeneratively, without scarring, GFs are relatively refractory to myofibroblast differentiation [11], making them especially suitable for achieving scarless, regenerative repair. Second, human gingival fibroblasts (HGFs) are highly accessible. Gingivae are one of the easiest tissues to extract for biopsy and may be isolated from donors with little discomfort. Indeed, they are frequently removed during routine dental procedures and are classified as biological waste [12]. Third, compared to other dental tissues, gingival tissue can be used to collect a significant number of fibroblasts and stem cells without requiring the irreversible loss of a tooth to access the pulp, periodontal ligament, or dental follicle [13].

Collectively, as they possess progenitor/stem cell-like differentiation capacities and are easily accessible, HGFs represent a promising, yet underappreciated, component of innovative tissue engineering strategies, in which scarless and regenerative repair is desired.

## 2. Gingival Healing Resembles Fetal Repair

### 2.1. Gingival Repair Possesses Fetal-like Collagen Organization

Fetal and gingival connective tissue share a similar structure. HGFs exhibit additional fetal-like features in terms of matrix synthesis, migratory activity, and proliferative potential [9]. Fetal wound healing is characterized by an increased ratio of type III to type I collagen, in which collagen is rapidly deposited in a fine reticular pattern with less interfiber space, while scars in postnatal wound show thicker collagen fibers with larger interfiber spaces that provide more rigidity to the healed tissue [14]. The fine reticular pattern of newly formed immature type III collagen in fetal wounds is thought to contribute generating a microenvironment that is refractory to the mechanical stress that promotes myofibroblast differentiation in adult scarring wound healing [15]. Similarly, treating murine excisional skin wounds with HGFs showed enhanced collagen III production at an early stage with little effect on collagen I, resulting in a higher ratio of collagen III/I, which indicates a more rapid and possibly fetal-like scarless healing response [16]. In addition, HGFs migrate more rapidly on collagen III compared to collagen I, which contributes to their lower adhesion and rapid invasion to the wound site to initiate tissue remodeling [17]. The migratory behavior of HGFs has been found to be in a fetal-like fashion by showing a fetal-like cell density migration index, as they show an elevated migratory activity, regardless of the cell density, compared to adult skin fibroblasts [9].

Scarless fetal wounds also are associated with an increased ratio of the molecules involved in ECM remodeling, the matrix metalloproteinase (MMPs), to tissue-derived inhibitors of metalloproteinase (TIMPs), compared to adult skin [18]. Moreover, HGFs express higher levels of MMPs, especially MMP-1, MMP-3, and MMP-10, compared to skin fibroblasts, although both cell types express similar levels of TIMPs [19]. This high expression level of MMPs in both gingival and fetal wounds is believed to result in an ECM organization that promotes cell migration [20].

### 2.2. Gingival Wounds Have Fetal-like Immunomodulatory Properties

In contrast to non-gingival adult wounds, gingival repair resembles that of fetal wounds by displaying a substantially less intense inflammatory responses and an initial inflammatory response of shorter duration [21,22]. Specifically, oral mucosal wounds exhibit a fetal-like inflammatory signature, including significantly decreased IL-6 production and fewer number of macrophages, mast cells, neutrophils, and T cells compared to adult dermal wounds [23,24,25]. In addition, as discussed above, HGFs express a high level of MMPs, compared to adult skin fibroblasts [18,19]. MMPs, particularly MMP-3, have potent anti-inflammatory effects by suppressing macrophage activation and inhibiting IL-6 expression [26,27]. Recently, HGFs have been directly shown to possess a fetal-like immunomodulatory function in a mouse skin wound healing model [16]. In this study, mouse wounds treated with either HGFs or HGF-derived conditioned media showed enhanced healing and a milder inflammation at day 3 and day 7 post-wounding, as visualized by a reduction in both neutrophil numbers and of TNFα production, at levels comparable to those present in scarless fetal wounds [16]. Overall, the immunomodulatory properties of HGFs—the suppression of the production of pro-inflammatory cells and cytokines and the promotion of tissue homeostasis/repair—result in the exceptional healing capacities of the gingiva that resembles that of the fetus.

### 2.3. Gingival Wounds Are Scarless and Possess Fetal-like Regenerative Capacity

Fetal wounds, within the first two trimesters in humans, possess the ability to heal faster, without scarring, compared to adult skin wounds [15]. Fetal wounds are characterized by reduced contraction, mirrored by the absence of contractile α-smooth muscle actin (SMA)-expressing myofibroblasts, compared to adult wounds [28]. Likewise, gingiva heal without scarring, and fibrotic responses in the gingiva are characterized by hyperproliferation, but not scar tissue formation [29]. Cultured HGFs are relatively refractory to myofibroblast differentiation in response to mechanical strain and the fibrogenic cytokine TGF-beta1 [11,19,30,31]. In vivo, the absence of myofibroblasts in gingival healing has been demonstrated using a rat gingivectomy model, where the α-SMA-expressing cells were present adjacent to blood vessels, but not embedded within connective tissue, i.e., they were more likely to be smooth muscle cells, but not myofibroblasts. Furthermore, the same study showed that the matricellular protein periostin promotes myofibroblast differentiation of human dermal fibroblasts, but not HGFs cultured in vitro [32]. Similarly, although other oral mucosal tissue can show partially fibrotic healing, myofibroblast differentiation is not apparent in human gingival wounds [33]. Intriguingly, a recent in vivo study showed that delivering either HGFs, or conditioned media derived from cultured HGFs, accelerated healing of murine excisional skin wounds, as visualized by elevated in angiogenesis and collagen deposition, without causing increased myofibroblast differentiation [16]. Collectively, these data are consistent with the notion that gingival healing resembles regenerative and scarless fetal repair (Table 1).

## 3. Gingival Mesenchymal Stem Cells (GMSCs)

### 3.1. GMSCs Are Pluripotent

**Figure 1:**
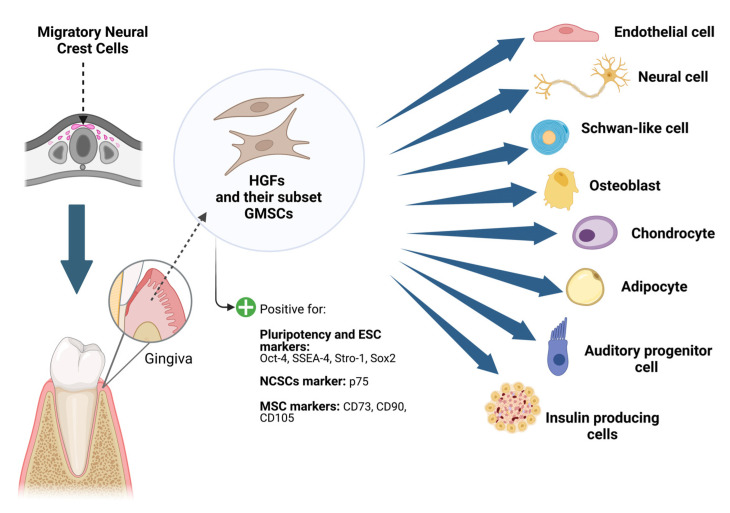
Pluripotency of human gingival fibroblasts (HGFs) and their derived subset gingival mesenchymal stem cells (GMSCs). Figure generated using Biorender.

The gingival tissue’s exceptional capacity for regenerative repair a priori suggests the existence of a substantial resident stem cell population. Indeed, resident gingival fibroblast subpopulations possessing adult stem cell-like properties are termed gingival mesenchymal stem cells (GMSCs) [10] (Figure 1). These properties include a colony-forming capacity, in vitro proliferation as plastic-adherent cells with fibroblast-like morphology, self-renewal, and immunomodulatory and anti-inflammatory functions, as well as multipotent differentiation (Figure 1). Distinct subpopulations of HGFs co-express stem/progenitor markers such as stem cell factor (SCF) and its receptor c-kit, which encourage cell division, proliferation, and recruitment of progenitor cells in various biologic systems [34,35]. Additionally, cell populations in the gingival lamina propria express Oct-4, SSEA-4, and Stro-1, the pluripotency-related markers [35]. This was supported when a further study found that the gingival lamina propria has a population of cells that are positive for the ESC transcription factors Oct-4 and Sox2, as well as low affinity neurotrophin receptor (p75), a marker of neural crest stem cells [36]. Recently, the MSC precursor markers CD73, CD90, and CD105 were expressed by outgrown MSCs obtained from gingival tissues [37]. These results support the existence of stem cells in the human gingiva as well as the stemness characteristics of HGFs. However, another study showed that, compared to human foreskin fibroblasts and human dental pulp stem cells (hDPSCs), HGFs displayed significantly lower levels of MSC markers, CD73, CD90, and CD105 [38]. Nonetheless, these data collectively suggest that a subset of HGFs express progenitor cell markers, making it likely that the gingiva could be an excellent source of mesenchymal progenitor cells.

Consistent with this notion, HGFs, when cultured on chitosan membranes, form spheroids that are multipotent as they express neural crest marker genes and display multilineage differentiation potential [39]. The differentiation potential toward endothelial, neurogenic, osteogenic, adipogenic, or chondrogenic differentiation lineages is enhanced when using a spheroid, compared to a monolayer, culture [40,41]. It is interesting to note that spheroids derived from dermal fibroblasts also express neural crest markers and are pluripotent [42]. Dermal-derived spheroids differentiate into myofibroblasts [42]; it is tempting to speculate the gingival-derived fibroblasts may be unable to do so, as myofibroblasts are largely absent from gingival wounds.

### 3.2. GMSCs Have Potent Immunomodulatory Properties

In contrast to MSCs derived from other tissues, which decline in quality and quantity with ageing, GMSCs maintain both immunoregulative and regenerative potential. This feature was demonstrated when human GMSCs reproducibly and independently of the donor’s age suppressed the in vitro proliferation of peripheral blood mononuclear cells (PBMNCs) and in vivo immunogenicity of LPS in a mouse model of acute lung injury [43]. Also, in a mouse model of hyperlipidemia with periodontitis, GMSCs significantly reduce the serum levels of pro-inflammatory cytokines IL-6 and TNF-α, while increasing the anti-inflammatory cytokine IL-10 and improving periodontal tissue regeneration [44]. Recently, GMSCs showed their ability to limit neutrophil infiltration in bleomycin-induced pulmonary fibrosis in mice [45].

In addition to their ability to promote Treg infiltration, many studies demonstrated GMSCs’ capability to suppress T helper cell differentiation, including Th1, Th2, and Th17, and to inhibit B cell proliferation and plasma cell differentiation via the CD39/CD73 pathway. For example, human GMSCs showed effective immunosuppressive functions in a mouse model of collagen-induced arthritis (CIA) by significantly decreasing the severity of arthritis through downregulating the inflammatory cytokine (IFN-γ, IL-17A), as well as upregulating CD4+CD39+Foxp3+ Treg cells and suppressing the differentiation of mouse T helper cells Th1, Th2, Th17 through CD39/CD73 signals [46]. Along with that, by regulating T effector cells via the CD39/CD73 pathway and drastically reducing murine CD4+ T cell differentiation into Th1 or Th17, human GMSCs alleviated the diabetic condition in streptozotocin (STZ)-induced type 1 diabetes (T1DM) mice [47]. In addition, injection of human GMSCs into a murine model of acute graft-versus-host disease (GVHD) stabilized Foxp3 expression, enhanced the differentiation and suppressive function of CD4+ iTreg cells, as well as reduced pro-inflammatory cytokine production such as IFN-, IL-17, IL-4, and TNF-α [48]. Additionally, a later study showed that adoptively transferred human GMSCs into lupus nephritis mice migrate to the kidney to reduce glomerulonephritis and proteinuria. This was accomplished by GMSCs’ capacity to inhibit B cell proliferation and plasma cell differentiation via the CD39/CD73 signaling pathway, downregulate the immune response of T cells, lower the frequency of Th2 and Th17, and decrease the levels of IgG and IgM autoantibodies [49]. In addition to their ability to suppress inflammatory cells, GMSCs have also recently showed the capacity to hinder the proliferation of rheumatoid arthritis fibroblast-like synoviocytes through the CD39/CD73 signaling pathway in vitro, as well as decreased pro-inflammatory cytokines production. This function helped delay arthritis onset and suppressed bone and cartilage destruction when human GMSCs injected into a CIA mouse model [50].

In addition, human GMSCs induce multiple alterations in the phenotype of human macrophages. Specifically, GMSCs, particularly exosomes derived from GMSCs, convert macrophages to an anti-inflammatory M2 phenotype, promote phagocytosis, and increase expression of the anti-inflammatory cytokine IL-10, but reduce expression of the proinflammatory cytokine tumor necrosis factor (TNF-α), and thus alleviate periodontitis and bone loss [51,52,53,54]. In a mouse excisional skin wound model, GMSC-mediated polarization of M2 macrophages also accelerates healing by promoting the formation of highly organized and densely packed collagen fibers, neovascularization and re-epithelization [55]. At least one study showed that the ability of HGFs to promote the M2-like phenotype was superior to that of bone marrow MSCs [56]. That GMSCs promote the M2 macrophage phenotype was also seen in rat models of erectile dysfunction [57] and axonal regeneration [58].

Collectively, the above studies support the concept that GMSCs possess substantial immunosuppressive effects that would be expected to assist in the treatment of autoimmune and inflammatory diseases.

## 4. Induced Pluripotent Stem Cells (GF-iPSCs) Can Be Derived from GFs

Direct reprogramming of adult somatic cells into Induced Pluripotent Stem Cells (iPSCs) represents a revolutionary technology that boosted the regenerative therapeutics and differentiation potential. Reprogramming is conducted by induced expression of transcription factors including Oct4, Sox2, NANOG, and LIN28 in somatic cells. Since the iPSCs have similar properties to human embryonic stem (ES) cells, rejection of non-autologous cells, short lifespan, as well as ethical concerns related to the ES cells can be avoided [59].

GFs are an attractive source for iPSCs due to their readily accessibility and reprogramming capability. In an early study, in vitro mouse GFs showed higher reprogramming efficiency into iPSCs and increased proliferative capacity compared to the tail-tip fibroblasts, when they were reprogrammed via the introduction of four factors (Oct3/4, Sox2, Klf4, and c-Myc) or three factors (Oct3/4, Sox2, and Klf4, even without c-Myc), without drug selection [60]. Primary HGFs can also be transformed into HGF-derived iPSCs (GF-iPSCs) by overexpressing the ES cell-specific genes NANOG, REX1, TERT, OCT3/4, and SOX2. The resultant HGF-iPSC were able to differentiate into derivatives of the three germ layers in vivo [60]. A subsequent investigation confirmed these results, showing that HGFs transduced with Oct3/4, Sox2, Klf4, and c-Myc displayed pluripotency, produced ES cell-like colonies, and expressed the human ES cell-surface antigens SSEA3, SSEA4, GCTM-2, TG30 (CD9), and Tra-1-60, as well as the human ES cell marker genes OCT4, NANOG, and GDF3 [61]. That HGFs are capable of expressing the iPSC-associated genes is perhaps unsurprising, given that a recent study assessing expression of MSC/stemness markers in healthy gingival tissues revealed a high abundance of cells expressing Sox2, c-Myc, and KLF4 [62]. Intriguingly, HGF-iPSCs can not only be generated using standard viral transduction methods, but also using episomal plasmid vectors [63]. These integration-free HGF-iPSCs showed strong expression of the pluripotency markers Oct4, Tra181, Nanog, and SSEA-4, and high periodontal differentiation potential by expressing periodontal tissue markers associated with bone, periodontal ligament, and cementum, after in vitro treatment with enamel matrix derivative or growth/differentiation factor-5 [63]. Collectively, the available data suggest that GFs may represent a promising initial material for generating iPSCs suitable for use in regenerative medicine.

## 5. Translational Relevance of Gingiva-Derived Mesenchymal Progenitor Cells

### 5.1. Angiogenesis and Neovascularization

One of the challenges of tissue engineering and regeneration is to achieve sufficient in vitro pre-vascularization and in vivo neovascularization to sustain and preserve the survival of implanted tissues to permit integration into the host tissue [64,65]. This neovascularization requires the proper orchestration of a variety of different cell types, growth factors, and extracellular matrix components [66]. HGFs significantly interact with endothelial cells and surrounding microenvironment, which enhance the angiogenesis and vascularization necessary for tissue regeneration [67].

HGFs have been successfully used in generating a highly vascularized scaffold for tissue regeneration applications [68,69]. In one study, an in vitro perfusion system of a co-culture of HGFs with human umbilical vein endothelial cells (HUVECs) in a degradable/polar/hydrophobic/ionic polyurethane (D-PHI) hydrogel scaffold was used to facilitate regeneration of atrophied gingiva [68]. The greater ratio of HGFs to HUVECs in this system displayed more enhanced cell growth and increased production of angiogenic factors, including vascular endothelial growth factor (VEGF) and fibroblast growth factor (FGF)-2, and, importantly, decreased myofibroblast differentiation [68]. In another study, using a 3D coculture system of HUVECs and different fibroblasts, HUVECs showed greater ability to organize and form denser and more interconnected 3D networks with HGF compared human dermal fibroblasts or stem cells from the apical papilla [69]. Moreover, HGFs possess high perfusion capacity by attracting blood vessels and promoting neovascularization. New vessels grew on the surfaces of HGF-seeded silk fibroin scaffold which was implanted into the chicken embryonic chorioallantoic membrane (CAM) of fertilized chicken eggs [70]. The growing vessels occupied more scaffold areas in the samples seeded with HGFs compared to those seeded with human dental pulp stem cells (HDPSCs). However, there were no statistically significant differences between HDPSCs and HGFs in terms of perfusion capacity.

### 5.2. Neurogenesis

Neural tissue is characterized by a low capacity to repair itself after injury [71]. As discussed above, gingivae contain cells that express neural crest- and mesoderm-MSCs markers [8,72], implying that the gingiva could be a source for neural crest (NC)-like stem/progenitor cells (NCSCs) [73]. Specifically, HGFs can be cultured as neurospheres and express NC markers such as nestin and Sox9 and can also differentiate toward a neuronal lineage [73]. Indeed, in such studies, a higher expression of neurogenic markers and a greater yield of neuron-like cells are achieved using GMSCs compared to using stem cells from other sources, including apical papilla (SCAP), bone marrow MSCs, foreskin, and dermal fibroblasts [74,75]. In addition, differentiation of GMSCs neurospheres produced functional neural cells are revealed by detectable electrophysiological function and action potential [74].

In addition to the powerful neural differentiation potential of GMSCs, their contribution to scarless healing points to the possibility of using GMSCs to overcome the scar formation that leads to axonal damage, limiting functional recovery after nerve injury [40]. Human gingival derived neuronal stem cells (GNSCs) generated by neuronal induction of GMSCs, in combination with injectable caffeic acid bioconjugated hydrogel, helped restore damaged spinal cords in a rat model by promoting axonal growth, remyelination, and the formation of new synaptic vesicles [40]. Likewise, transplanting human GMSCs alleviated cavernous nerve injury-induced erectile dysfunction in a rat model by restoring of neuronal nitric oxide synthase expression, and increased nerve growth factor and myelin basic protein levels in major pelvic ganglia [57]. Furthermore, human GMSCs showed the ability to be converted into Schwann-like cells and represented a neurotrophic role in promoting facial nerve regeneration in a rat model [76], as well as enhancing axonal regeneration and functional recovery in crush-injured rat sciatic nerves through non-genetic methods using neuronal or Schwann cell induction media [58]. According to these studies, human GMSCs may be a useful cell source for regenerative therapies in peripheral nerve damage.

Recently, human GMSCs have been demonstrated as a promising source for cell-based therapy to restore neural damage associated with neurodegenerative diseases such as Parkinson’s disease (PD). In the 6-Hydroxydopamine-induced PD rat, human GMSCs helped improve movement and misaligned behavior, upregulated neural regeneration-related molecules, and inhibited the activation of astrocytes and microglia in PD. Also, GMSCs showed a neuroprotective effect by reducing mitochondrial membrane potential damage and reactive oxygen species accumulation [77].

Interestingly, it has been reported that human GMSCs, following prolonged passaging, showed spontaneous differentiation into neural progenitor cells. At passage 41, GMSCs showed a change in morphology from typical fibroblasts into sphere-shaped cells with extending processes and exhibited de novo expression of neural precursor genes, such as NRN1, PHOX2B, VANGL2, and NTRK3. The findings indicate that HGMSCs might help provide a novel in vitro tool for neural disease modelling [78].

### 5.3. Osteogenesis

HGFs can differentiate toward an osteogenic lineage in response to dexamethasone (dex), which induced expression of osteopontin, osteocalcin, alkaline phosphatase (ALP), and synthesis of a calcified matrix [79]. HGFs respond to being cultured in an osteogenic medium by expressing the osteoblast-specific transcription factors Runt-related transcription factor 2 (Runx2) and Osterix, and the reprogramming factors Oct4 and L-Myc, in addition to the production of osteocalcin, ALP, and calcified bone matrix [80]. The resultant osteoblast-like cells, when transplanted into mice, can repair bone damage [80]. Furthermore, when injected into mice, a collagen hydrogel containing bone morphogenic protein (BMP)-transduced HGFs can promote the development of bone marrow and trabeculae [81]. In addition, HGF-iPSCs inserted in nanohydroxyapatite (nHA)/chitosan/gelatin scaffolds showed enhanced osteogenic differentiation and osteogenic marker expression in vitro and, in vivo, produced bone-like tissue with increased osteogenic marker expression of RUNX-2 and OCN [82]. Finally, exosomes derived from human GMSCs promote osteogenic differentiation and migration of pre-osteoblasts in vitro, which was evidenced by enhanced ALP activity and elevated expression of osteogenic differentiation-related genes, such as BMP2, OCN, RUNX2 and OPN [13].

In addition, HGFs and GMSCs can suppress osteoclastogenesis of pre-osteoblasts, suggesting that these cells could represent a treatment for diseases characterized by bone destruction, such as rheumatoid arthritis and periodontitis [13,83,84]. By inhibiting osteoclast formation, including the expression of RANKL, human GMSCs significantly reduced bone erosion in mice subjected to collagen-induced model of arthritis [83]. In addition, HGFs inhibited osteoclastogenesis and alveolar bone destruction in the LPS-induced model of periodontitis [84]. Finally, when co-cultured with inflammatory cells, HGFs inhibit MMP production [85]. Collectively, these data suggest that HGFs have the potential to treat rheumatological disorders.

### 5.4. Chondrogenesis

Both HGFs and their subset GMSCs showed chondrogenic potential in many studies. For example, HGFs cultured on chitosan membranes showed spheroid formation, as well as enhanced chondrogenic differentiation potential and expression of chondrogenesis related genes when induced with chondrogenesis induction medium [86]. In addition, GMSCs showed chondrogenic differentiation potential, which was evident by presence of sulfated proteoglycans after chondrogenic induction [74]. As further evidence of the chondrogenic differentiation capacity of GMSCs, it has been shown that chondrogenic-induced GMSCs generated cartilage balls [57].

### 5.5. Dental and Periodontal Tissue

HGFs have shown immense capabilities in the regeneration of both hard and soft dental and periodontal tissues, which highlights their potential as a valuable tool in regenerative dentistry. HGFs have been used as seed cells in a sandwich tissue-engineered construct, and due to the significant role of HGFs in osteogenesis and mineralization, periodontal defects were completely repaired in dogs [87]. Moreover, GMSCs differentiate into osteoblasts and develop mineralization nodules in vitro. In comparison to periodontal ligament MSCs (PDLMSCs), the proliferation potential of GMSCs was greater. However, PDLMSCs demonstrated more osteogenic potential with stronger mineral nodules and greater expression of osteogenic genes like ALP and COL I. Despite this, human GMSCs still possess osteogenic potential, and their abundant availability and easy accessibility compared to PDLMSCs make them a potent source of stem cells for cell-based periodontal and bone therapies [37]. In addition, systematically transplanted GMSCs in mice with periodontitis showed the ability to home to periodontal defect sites, decrease alveolar bone loss, and promote periodontal tissue regeneration [88].

Furthermore, HGFs influence and enhance the regeneration of dental pulp. Conditioned media derived from cultured HGFs (HGF-CMs) promote dental pulp regeneration through improving the proliferation and migration capacity of dental pulp stem cells and enhancing the differentiation of odontoblasts, associated with the presence of ECM proteins in HGF-CMs, such as collagen and laminin [89]. Thus, HGFs, and their subpopulation GMSCs, represent a promising tool in the field of regenerative dentistry.

### 5.6. Fibrosis

HGFs promote connective tissue regeneration. When used to treat mouse skin damaged by radiation burns, HGFs stimulate the regeneration of hair follicles, epidermis, and dermis [56]. Similar results were seen using GMSCs in a murine skin transplantation model [90]. Mouse GMSC may promote scarless repair, as they suppress bleomycin-induced pulmonary fibrosis by inhibiting the expression of the profibrotic and proinflammatory genes [45]. Moreover, HGF-derived spheroids, when incorporated into a fibrous protein-based hydrogel, express progenitor markers, such as Sox2 and nestin, and continue to proliferate and synthesize/turnover ECM components, suggesting that these hydrogels could be used in the future to as multipotential building blocks for connective tissue regeneration [91]. Conversely, use of dermal-derived spheroids may result in myofibroblast differentiation, and hence fibrosis, through the action of the matricellular protein CCN2 [42,92]. Mesenchymal stem cell transplantation has emerged as a potentially disease-modifying treatment for the fibrotic autoimmune disease systemic sclerosis (scleroderma) [93]; perhaps the use of GMSCs for this application is warranted.

### 5.7. Hearing Loss

As a novel and convenient source of stem cells with high multipotent differentiation potential, human GMSCs were used for the treatment of sensorineural hearing loss in mice by differentiation into auditory progenitor cells within an engineered microenvironment that contained a three-dimensional alginate–Matrigel composite hydrogel scaffold in addition to the growth factors epidermal growth factor, insulin-like growth factor and basic fibroblast growth factor [94].

### 5.8. Diabetes

Human GMSCs have recently demonstrated the ability to differentiate into Insulin Producing Cell Clusters (IPCCs) and to express pancreatic markers such as insulin, pdx1, glucagon, GLUT4, and GLUT2 in vitro [95]. As a result, the GMSCs provide an attractive, approachable, and non-invasive model source for diabetes research as well as for autologous stem cells transplantation in type 1 diabetic patients without the requirement for immunosuppression.

## 6. Limitations and Future Directions

Despite the potential of HGFs for regenerative medicine, there are several limitations and challenges that must be addressed before they can be widely used in clinical applications. One of the major limitations is the heterogeneity of HGFs, which can affect their regenerative potential [12]. These issues may be partially attributed to the existence of many fibroblast-like cells in connective tissues and fibroblast cultures, such as myofibroblasts, fibrocytes, pericytes, and mesenchymal stem cells (MSCs). For example, in vivo, stem cell factor (SCF) and its receptor c-kit are expressed in different subsets of HGFs [35]. In addition, the phenotype and behavior of fibroblasts may also alter because of aging or differentiation [96,97], and their developmental origin (e.g., mesodermal and neural crest origin) [72]. The lack of standardized protocols for HGFs isolation and characterization is another challenge that needs to be addressed as the different methods to isolate and propagate cells from the tissue biopsy may result in the selection of functionally distinct fibroblast population [98]. In addition, the crucial MSC marker STRO-1 as well as the transcription factors OCT-4, NANOG, and NESTIN gradually decreased in HGMSCs with increasing cell passaging. However, STRO-1 may be a helpful marker for isolation of undifferentiated MSCs from gingiva for differentiation toward multiple lineages [99]. Moreover, HGMSCs displayed decreased in vitro adipogenesis as well as in vitro and in vivo osteogenesis with increasing age of the donor. However, in vitro neurogenesis remained unaffected [43]. Despite the promising capabilities of the HGF-iPSCs in regenerative applications, teratoma formation remains the main challenge associated with iPSCs [100]. It is unclear if similar issues exist when HGF-iPSCs are used. However, growing cells as spheroids in stem cell media or in environments that encourage plasticity by promoting the formation of pluripotent mesenchymal progenitor cells (e.g., culturing as neurospheres which express markers such as Sox2 or nestin) might address this issue. Also, the utilization of 3D culture systems, such as hydrogels, may facilitate the retention and acquisition of stemness and the integration of differentiated tissue. Finally, studies examining the role of epigenetics (i.e., the contribution of deoxyribonucleic acid (DNA) methylation, histone modification, and/or noncoding RNAs) in modulating behavior of dental stem cells might be informative in understanding how to control gingival stem cell plasticity [101].

Relatively few manuscripts have explored the utility of GFs in tissue engineering applications. Our hope is that our review will inspire these efforts.

## 7. Conclusions

In conclusion, HGFs have emerged as a promising cell source due to their accessibility, multipotency, and immunomodulatory properties. HGFs have exhibited excellent biocompatibility and low immunogenicity, making them attractive candidates for clinical applications. Moreover, the use of HGFs in combination with biomaterials, scaffolds, and bioactive molecules has further enhanced their regenerative potential and provided a platform for targeted and controlled tissue engineering strategies. Addressing the limitations and optimizing the protocols for their isolation, expansion, and differentiation will be crucial for their successful translation into therapeutic applications. With ongoing advancements and interdisciplinary collaborations, HGFs hold great promise for the development of innovative regenerative therapies, bringing us closer to a future where tissue repair and regeneration, including the treatment of fibrotic diseases such as scleroderma, can be achieved with enhanced efficiency and positive clinical outcomes.

## Figures and Tables

**Table 1:** Similarities between scarless fetal and gingival healing.

Similarities		References
Collagen organization	Increased ratio of type III to type I collagen. Collagen fibers are organized in fine reticular pattern with less interfiber spaces.	[14,15,16]
ECM remodeling	Increased ratio of MMPs to TIMPs which promotes ECM remodeling.	[18,19,20]
Migratory behavior of fibroblasts	Low adhesion and rapid invasion to the wound site. Fetal-like cell density migration index.	[9,17]
Immunomodulatory properties	Mild and relatively short inflammatory onset. Reduced production of pro-inflammatory cytokine IL-6. Fewer number of inflammatory cells. Anti-inflammatory condition due to a high level of MMPs.	[21,22,23,24,25,26,27]
Reduced contraction	Absence of myofibroblasts.	[28,29,33]
